# Dataset of dust mass accumulation rates for the loess-palaeosol sequences from the Carpathian Basin

**DOI:** 10.1016/j.dib.2022.108555

**Published:** 2022-08-28

**Authors:** Zoran M. Perić, Thomas Stevens, Igor Obreht, Slobodan B. Marković

**Affiliations:** a Department of Geology, Lund Luminescence Laboratory, Lund University, Sölvegatan 12, Lund SE-223 62, Sweden; b Department of Earth Sciences, Uppsala University, Villavägen 16, Uppsala 75236, Sweden; c Organic Geochemistry Group, MARUM, Center for Marine Environmental Sciences and Department of Geosciences, University of Bremen, Leobener Str. 8, Bremen 28359, Germany; d Chair of Physical Geography, Faculty of Science and Mathematics, University of Novi Sad, Trg Dositeja Obradovića 3, Novi Sad 21000, Serbia

**Keywords:** Age-depth model, Loess-palaeosol sequences, Atmospheric dust activity, Sedimentation rates, MAR, R.bacon

## Abstract

In this article, a dataset of age-depth modelling data, sedimentation rates and dust mass accumulation rates (MAR) from four loess-palaeosol sequences from the Carpathian Basin is presented. The dataset is related to the article “Detailed luminescence dating of dust mass accumulation rates over the last two glacial-interglacial cycles from the Irig loess-palaeosol sequence, Carpathian Basin”, published in the journal Global and Planetary Change by Perić et al. [1].

In the dataset, luminescence ages from the loess sites Irig, Nosak, Stari Slankamen and Crvenka were modeled using the r.bacon software after which the dust mass accumulation rates were calculated. For a more realistic representation the MARs were subsequently smoothed using the SigmaPlot software. For all sites, minimum, maximum, median and mean values for the modelled ages and accumulation rates are calculated and presented.

**Specifications Table**

**Table:** 

Subject	Earth and Planetary Sciences
Specific subject area	Earth-Surface Processes, Stratigraphy
Type of data	Figure Microsoft Excel
How the data were acquired	For the luminescence dating of the Irig LPS data was acquired through sampling and laboratory measurements. The luminescence ages were published in Perić et al. [1]. For the Nosak, Stari Slankamen and Crvenka LPS, data was collected, analyzed and modelled from published papers of Perić et al., Murray et al. and Stevens et al., respectively [2], [3], [4]. Software used for data modelling: R.Bacon version 2.5.8, SigmaPlot v. 11.0.
Data format	Raw data Analyzed data Modelled data Smoothed data
Description of data collection	The original data were acquired using literature review and determining loess-palaeosol sequences with the most detailed luminescence-based chronologies in the Carpathian Basin. The luminescence ages were subsequently modelled using the r.bacon software after which the dust mass accumulation rates were calculated.
Data source location	• The luminescence dating samples for the Irig LPS used for the age depth modelling were collected in north Serbia (45°05`N; 19° 52′E). • For the Nosak LPS, published luminescence ages were used from Perić et al. [2]. • For the Stari Slankamen LPS, the luminescence ages were adopted from Murray et al. [3]. • For the Crvenka LPS, luminescence ages were used from the study of Stevens et al. [4].
Data accessibility	**Raw and modelled data:** Repository name: Mendeley Data Data identification number: https://10.17632/7cgxs47cjg.1 Direct URL to data: https://data.mendeley.com/datasets/7cgxs47cjg/1 **r.bacon settings:** https://github.com/ZoranPeric/r.bacon-settings/blob/main/r.bacon
Related research article	**For an article which has been accepted and is in press:** Z. M. Peric, T. Stevens, I. Obreht, U. Hambach, F. Lehmkuhl, S. B. Marković, Detailed Luminescence Dating of Dust Mass Accumulation Rates Over the Last Two Glacial-Interglacial Cycles from the Irig Loess-Palaeosol Sequence, Carpathian Basin. Glob. Planet. Change. (215) 103,895 [1]. https://doi.org/10.1016/j.gloplacha.2022.103895

## Value of the Data

• This article presents a unique set of calculated and modelled dust mass accumulation rates for four loess-palaeosol sequences from the Carpathian Basin with different geomorphological settings and distances from the main silt source.
• This dataset represents an examination of atmospheric mineral dust activity trends and provides an objective basis for understanding past environmental changes during glacial and interglacial periods.
• This dataset can be used in further research on atmospheric dust activity during the last two glacial-interglacial cycles and global as well as regional comparisons between different palaeoclimatic records.
• This data is of interest to scientists who study the environmental and climatic changes and atmospheric dust activity under glacial and interglacial conditions as well as for climate modelers.

## 1 Data Description

**Fig. 1:**
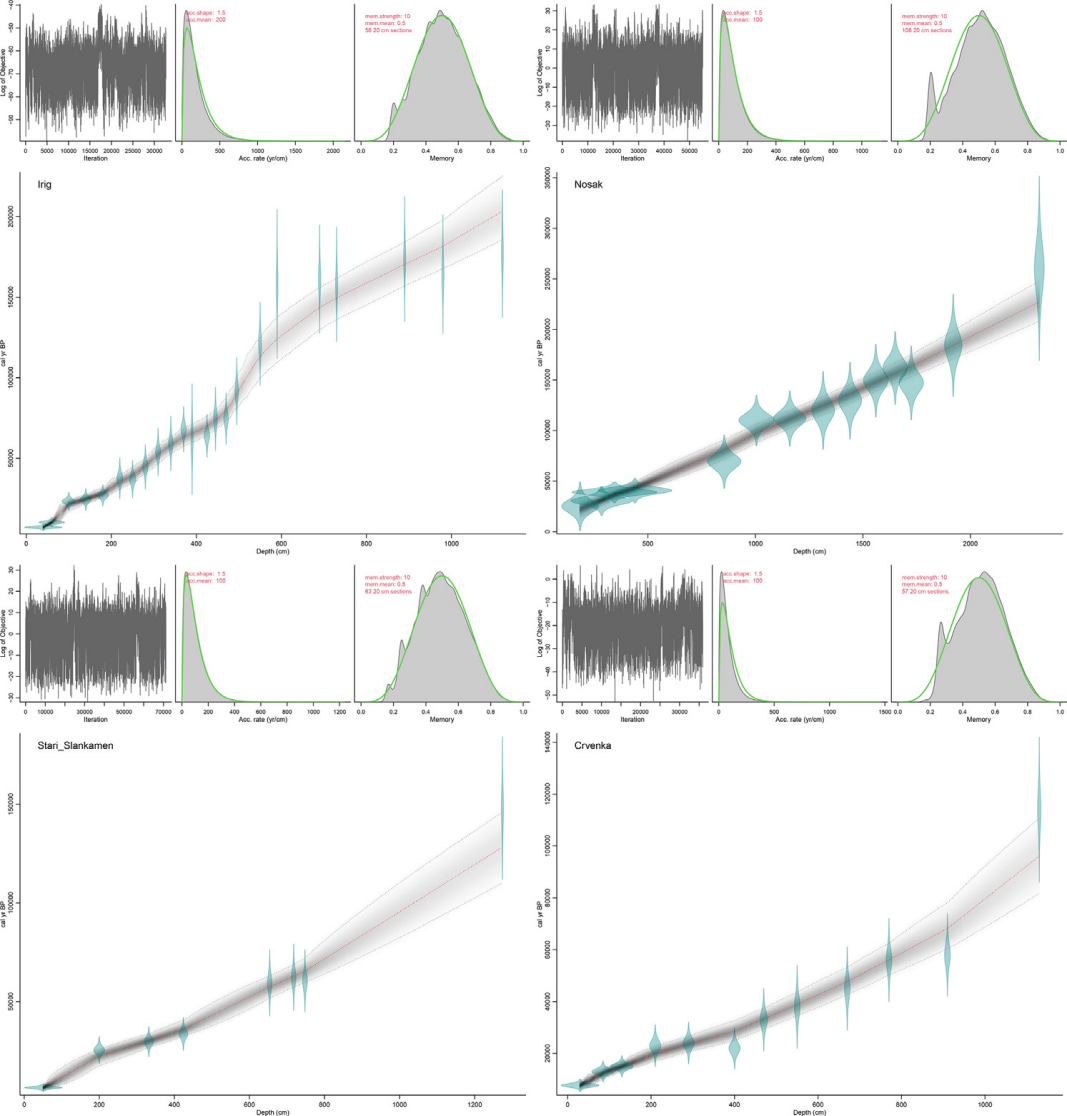
Bacon.r age models for the: (a) Irig; (b) Nosak; (c) Stari Slankamen and (d) Crvenka LPS. Top panel: Time series of the log-posterior for the sub-sampled MCMC (left), prior (green) and posterior (gray) for accumulation rates with priors acc.shape = 1.5 and acc.mean = 200 for Irig and 100 for Nosak, Stari Slankamen and Crvenka (center) and prior and posterior for the memory (right), mem.strength = 10 and mem.mean = 0.5.

**Fig. 2:**
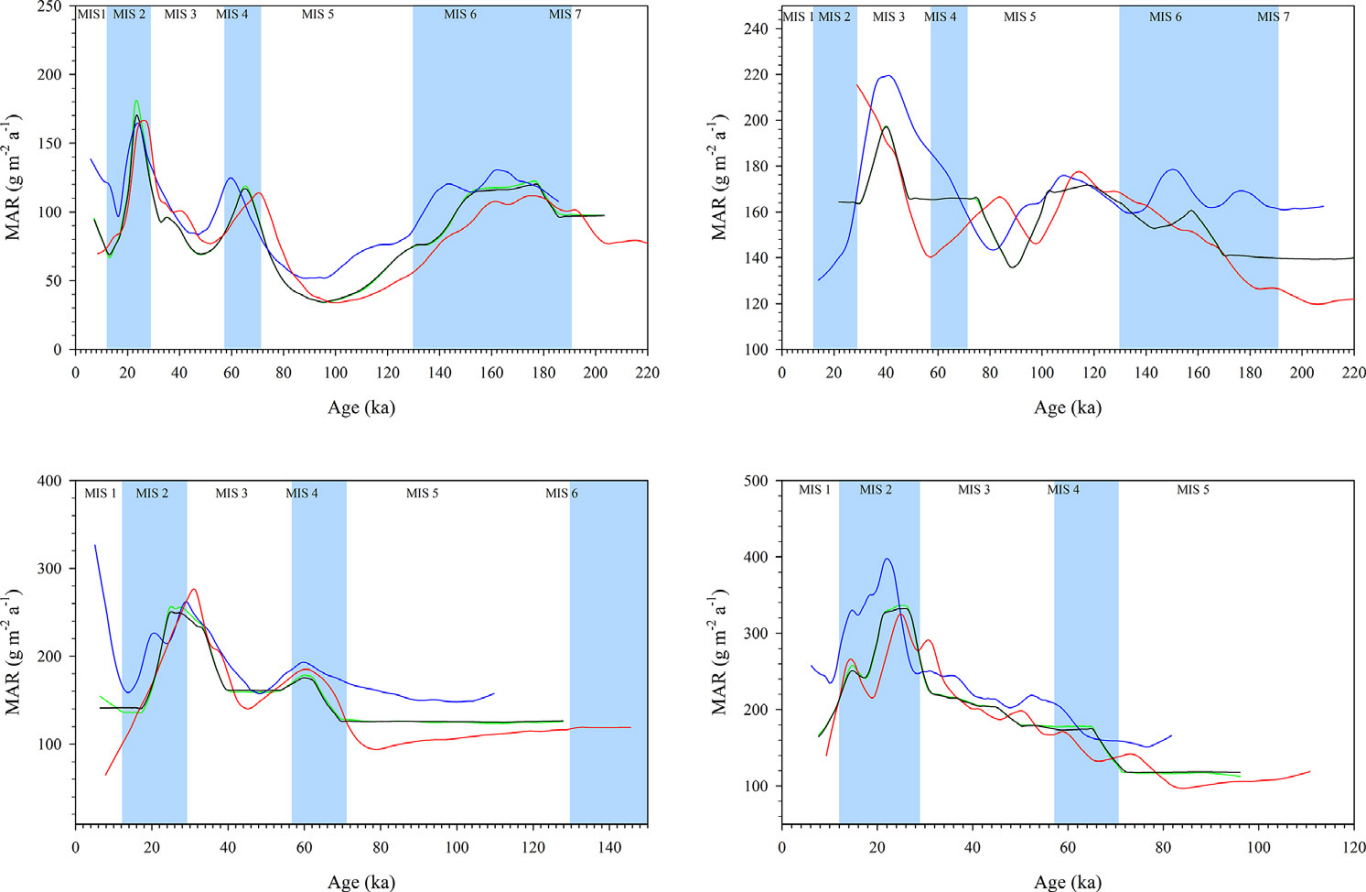
Dust Mass Accumulation Rate (MAR) as a function of age for the: (a) Irig; (b) Nosak; (c) Stari Slankamen and (d) Crvenka LPS. The blue curves present the MARs according to the minimum ages, the red curve shows the MARs for the maximum ages, the green curve represents the MARs for the median ages while the black curve shows the MARs according to the mean ages at a 95% confidence interval. For a more realistic representation the results were smoothed using the loess smoothing technique *via* the SigmaPlot v. 11.0 software.

The data presented in this article are related to the research article ‘Detailed luminescence dating of dust mass accumulation rates over the last two glacial-interglacial cycles from the Irig loess-palaeosol sequence, Carpathian Basin’ [1]. Fig. 1 displays the bacon.r age model for the: (a) Irig, (b) Nosak, (c) Stari Slankamen and (d) Crvenka LPS. The dataset displayed no inversions of the mean ages which is why the age-depth models were generated with only few resampling attempts and yielded stratigraphically consistent data. All age models were sensitive to luminescence age changes with depth which resulted in non-linear age-depth functions indicating variable sedimentation rates (SRs), at least within the uncertainty limits of the techniques. The age model for the Irig LPS (Fig. 1a) was constructed using 23 luminescence ages [1] and covers the period from 7 ± 5 to 177 ± 10 ka. The ages yield mean 95% confidence ranges of 20,503 years, minimum 2273 years at 45 cm and maximum 39,485 years at 1120 cm. 100% of the dates overlap with the age-depth model (95% ranges). The bacon.r age model for the Nosak LPS (Fig. 2b) was constructed using 14 luminescence ages [2] covering the time period from 25 ± 6 ka to 261 ± 23 ka. The ages yield mean 95% confidence ranges of 20,892 years, minimum 9285 years at 340 cm and maximum 39,559 years at 2320 cm. 100% of the dates overlap with the age-depth model (95% ranges). The Stari Slankamen bacon.r age model (Fig. 1c) was created using 8 luminescence ages [3] for the period from 6 ± 1 ka to 148 ± 9 ka. Based on the analysis, the mean age was calculated as 16,962 years with 95% confidence intervals, minimum 2777 years at 51 cm and maximum 35,817 years at 1271 cm. 100% of the dates overlap with the age-depth model (95% ranges). The bacon.r age model for the Crvenka LPS (Fig. 1d) is shown using 11 luminescence ages [4] spanning the period from 8 ± 1 ka to 114 ± 7 ka. According to the modelled ages, the mean 95% confidence interval is 11,878 years, with a minimum of 3050 years at 35 cm and a maximum of 29,057 years at 11,030 cm.

Fig. 2 displays the minimum, maximum, mean and median MARs for the (a) Irig, (b) Nosak, (c) Stari Slankamen and (d) Crvenka LPS. The MARs for the Irig LPS are presented for the period MIS 1-7 (Fig. 2a). The peak values were recorded during MIS 2 followed by MIS 4 and MIS 6. In general, the MARs show a high variability over the investigated time period. The minimum MAR values were recorded in MIS 5 and MIS 3, respectively. The mean calculated median and mean MARs were 97 and 95 g m^−2^ a^−1^, respectively.

The MARs calculated for the Nosak LPS are shown for the period MIS 1-7 (Fig. 2b). The MAR values peak during MIS 3 while the lowest values occurred during MIS 7 followed by MIS 5. At this site, we calculated a median MAR value of 163 m^−2^ a^−1^ and a mean of 158 m^−2^ a^−1^. For the Stari Slankamen LPS the MARs are presented for the period MIS 1-6 (Fig. 2c). The calculated median and mean MARs were 142 m^−2^ a^−1^ and 160 m^−2^ a^−1^, respectively. Peak values were recorded at the onset of MIS 2, while the lowest MARs were observed during MIS 5.

At the Crvenka LPS (investigated for the period MIS 1-5), a median value of 203 m^−2^ a^−1^ and a mean value of 208 m^−2^ a^−1^ were recorded (Fig. 2d). The peak MARs at this site occur during MIS 2 while the lowest values were recorded through MIS 5.

The raw as well as the maximum, minimum, mean and median modelled ages are available in Mendeley Data: https://data.mendeley.com/datasets/7cgxs47cjg/1 [5]. Also presented are the corresponding calculated SRs and MARs for the four investigated loess-palaeosol sequences. Additionally, the smoothed ages and MARs are also shown for each site.

The r.bacon settings used for the age-depth modelling of the luminescence ages are available in GitHub: https://github.com/ZoranPeric/r.bacon-settings/blob/main/r.bacon [6].

## 2 Experimental Design, Materials and Methods

The luminescence ages were modelled using the Bacon age-depth modelling software version 2.5.8 [7]. Using Bayesian statistics, Bacon combines numerical ages with prior information, including accumulation rate and variability (and changes in memory with depth).The accumulation rate is controlled by means of an autoregressive semiparametric model with gamma subdivisions through the sediment profile. A prior understanding of approximate accumulation rates can result in a more realistic model [7]. Models of accumulation and memory (autocorrelation) use gamma and beta distributions, and the latter determines how much the accumulation rate at a given depth varies with depth above it (memory effect). Section thickness affects the age-depth model's flexibility to some degree [8], which is obtained by performing millions of Markov Chain Monte Carlo (MCMC) iterations. For the age-depth modelling we used the following settings in r.bacon: d.by = 5, thick = 20, ssize = 8000. From the modelled ages, we calculated the sedimentation rates and the corresponding MARs. The MARs for each site were computed using the following formula:

MAR=SR×BD×feol

where, SR is the sedimentation rate (ma^−1^), BD is the dry bulk density and ƒeol is the fraction of the sediment that is of aeolian origin [8]. Since the origin of loess is assumed to be entirely aeolian we use the value 1 for our calculation. The used BD value was 1.5 g cm^−3^ according to Újvári et al. [9].

Finally, for a more realistic representation, the r.bacon modelled ages and corresponding MARs were loess smoothed using the SigmaPlot v. 11.0 software, with tricube weighting and polynomial regression (sampling proportion = 0.100; polynomial degree = 1).

## Ethics Statements

These data did not involve human subjects, animal experiments nor was it obtained from social media platforms and therefore does not contend with any ethical issue.

## CRediT authorship contribution statement

**Zoran M. Perić:** Data curation, Writing – original draft, Conceptualization, Methodology, Software, Visualization, Investigation, Writing – review & editing. **Thomas Stevens:** Writing – review & editing, Data curation, Writing – original draft. **Igor Obreht:** Writing – review & editing. **Slobodan B. Marković:** Supervision, Writing – review & editing.

## Declaration of Competing Interest

The authors declare that they have no known competing financial interests or personal relationships that could have appeared to influence the work reported in this paper.

## Data Availability

Age-depth models and dust-mass accumulation rates for loess-palaeosol sequences from the Carpathian Basin (Original data) (Mendeley Data).
